# Assessing the influence of building data choice on wildland–urban interface delineation in mainland Portugal

**DOI:** 10.1038/s41598-026-53590-5

**Published:** 2026-05-21

**Authors:** Bruno Barbosa, Sandra Oliveira, Caetano Mário, Jorge Rocha

**Affiliations:** 1 Centre of Geographical Studies, Institute of Geography and Spatial Planning, University of Lisbon, 1600-276 Lisbon, Portugal; 2 Associate Laboratory TERRA, Instituto Superior de Agronomia, Tapada da Ajuda, 1349-017 Lisbon, Portugal; 3 Direção-Geral do Território, Rua da Artilharia Um 107, 1099-052 Lisbon, Portugal; 4 Nova Information Management School (NOVA IMS) PT, Universidade Nova de Lisboa, Campus de Campolide, 1070-312 Lisbon, Portugal

**Keywords:** Wildland-urban interface, Building dataset, Geographic information system, Exposure, Environmental sciences, Environmental social sciences, Geography, Geography, Natural hazards

## Abstract

The delineation of the Wildland-Urban Interface (WUI) is fundamental for wildfire risk management, yet it is highly sensitive to the underlying building data used. Global building datasets offer unprecedented coverage but may introduce biases by including different types of built-up structures, potentially leading to an overestimation of exposure and a misallocation of critical resources. This study aims to map the WUI and assess wildfire exposure in mainland Portugal by comparing the efficacy of different building datasets: the official residential database (BGE21), Microsoft Building Footprints (MSB24), and the World Settlement Footprint (WSF19). We employed a point-based mapping methodology (100-m radius, > 6.17 buildings/km^2^) to classify WUI into *Intermix* and *Interface* zones. Our analysis revealed that the choice of dataset drastically alters WUI estimates. MSB24, which includes all structure types, identified 67% more buildings than the residential-focused BGE21, resulting in a 73% larger WUI area. Spatial agreement was low, with only 46% of the total WUI area being identified by all three datasets, falling to just 29% for the more vulnerable *Intermix* zones. While MSB24 showed high recall (0.97 *Intermix*, 0.99 *Interface*), its precision was low (0.43 *Intermix*, 0.67 *Interface*), confirming a significant overestimation of critical zones. Analysis of wildfire perimeters (2000–2023) showed that burned area within the WUI was disproportionately higher in years with smaller total fire extent (e.g., 2006, 2018) rather than in megafire years (e.g., 2003, 2017). We conclude that while global datasets like MSB24 are valuable for emergency response due to their high coverage, their use for preventive planning and resource allocation may cause inefficiencies. We recommend that local authorities prioritize validated residential data, like BGE21, for strategic wildfire prevention and mitigation planning in Portugal to ensure resources are targeted efficiently.

**Supplementary Information:**

The online version contains supplementary material available at 10.1038/s41598-026-53590-5.

## Introduction

In recent years, wildfire seasons in Europe have extended beyond the traditional summer period, notably, the 2023 fire season was one of the most severe since the 2000s, with over 500,000 hectares burned^1^. Furthermore, the continent has experienced three of its worst wildfire years in the past six years, based on burned area data since 1980^2^. Socio-economic dynamics, combined with climate change, play a significant role in intensifying wildfire behaviour and increasing the frequency of extreme events^3–6^. Every year, wildfires cause ecological degradation, destroy properties, infrastructures, agricultural lands, and pose a threat to human life through exposure to smoke and extreme heat^7,8^. For example, the fires that occurred in Pedrogão Grande (Portugal), in June 2017, burned more than 45,000 hectares, destroyed over 1,000 structures, and resulted in 66 fatalities^9^.

The Wildland-Urban Interface (WUI) represents areas where human structures are within or lie adjacent to fire-prone vegetation^10,11^. Each year, during the fire season, population in the WUI faces increased exposure to wildfires. WUI fires, which occur closer to human settlements and infrastructure, often result in significant destruction and economic losses^12–16^. Over recent decades, the expansion of these areas resulted in a larger population residing in fire-prone regions^14,17^. This trend has been observed in both global analyses^16,18^, and country-specific studies, such as in the United States^14^, Argentina^19^ and Portugal^20^, underscoring the consistent rise in WUI areas across scales. This growth is driven by two interconnected processes: the urban sprawl into natural areas^21^- which reduces the proximity between wildland vegetation and built-up areas^22^- and rural exodus, which contributes to the unmanaged regrowth of vegetation due to the abandonment of agricultural lands^14,23,24^. Human expansion in these zones triggers ecological disturbances, including the introduction of invasive species, changes in wildlife dynamics, pathogen spillover, and the conversion of land cover^25^. Furthermore, it increases the frequency of human-caused ignitions at the WUI^26–28^, particularly where human development encroaches on or intermixes with flammable vegetation that facilitates fire spread^11^.

The spatial distribution of the population is a key factor in identifying risk-prone areas, as it enables the analysis of communities’ vulnerability and exposure to different types of threats^29,30^. As zones where fatalities and economic losses concentrate, the WUI represent critical exposure points to severe wildfires, for both population and infrastructures^31^. When a wildfire reaches a WUI, the risk to population is influenced by multiple local factors, including the arrangement of buildings and their surroundings, the exposure of elements to fire, and the potential behaviour of the flames, as well as the presence (or absence) of basic prevention measures^10^. The vulnerability of buildings is strongly affected by housing patterns and location, with property losses being most likely in areas of low to intermediate structure density and regions with a frequent wildfire history^27,32^. Additionally, meteorological conditions, topography, socioeconomic factors, and the composition of vegetation play crucial roles in fire propagation within the WUI^12,33,34^. While land management strategies (even those not specifically designed for settlement protection) can reduce wildfire exposure in communities^33,35^, architectural design features that prevent ember intrusion are more critical to structure survival than defensible space, given the dominant role embers play in causing structure ignitions^9,15^.

The integration of high-resolution spatial data into risk analysis and disaster frameworks has transformed how communities assess exposure and vulnerability and how they allocate resources in crisis scenarios. In this context, satellite-derived building data play a crucial role in supporting decision-making processes in disaster risk management^36^. Microsoft has integrated advanced deep learning, computer vision, and artificial intelligence (AI) into its mapping systems, applying machine learning models to automatically extract building outlines from global satellite imagery^37^. These kinds of data underpin critical functions in risk management and disaster response, demonstrating their adaptability to diverse applications. For example, they enable population estimation^38^, exposure and vulnerability assessments, disaster response planning, and resource allocation^36,39^, as well as climate impact predictions^40^ and poverty estimation^41^. Buildings footprint data are a valuable resource for identifying Wildland-Urban Interfaces, as they provide consistent spatial coverage across large geographic areas. Internationally, mapping and analysing WUI patterns using these datasets has proven effective, enhancing the precision of exposure assessments and wildfire risk evaluations^42–47^.

Recent research highlights the importance of auditing geospatial datasets and AI workflows for biases in disaster risk management (DRM). As AI becomes more prevalent in DRM, concerns about fairness, accountability, and the disconnect between algorithm designers and affected communities are growing^48^. Studies have found biases in global building datasets against vulnerable populations, potentially affecting aid distribution^36^. Audits of flood vulnerability assessments revealed AI-introduced biases against certain housing types, which could lead to exclusion from risk management interventions^49^. Few studies have evaluated the quality of building footprints for WUI mapping. For instance, Bar-Massada^44^ and Carlson et al.^45^, relied on visual validation of the Microsoft Building footprint database in their study areas located in the United States by analysing high-resolution imagery. Although both studies reported high accuracy of the analysed data, their assessment was primarily limited to verifying building presence. It did not distinguish between residential and non-residential structures, underscoring the need for further targeted research in this area.

This study aims to map the WUI and assess wildfire exposure in mainland Portugal by using and comparing different building location datasets. Since the extreme wildfires occurred in the country in 2017, the legal and regulatory framework regarding wildfire management was modified; in this context, a new Integrated Rural Fire Management System is being implemented^50^, with particular emphasis on the protection of human settlements and the design of preventive and mitigation measures that require the mapping of buildings in their application. Within this scope, we conducted a comparative analysis between the WUI created with the data from the official Census database from Statistics Portugal and the Microsoft Building Footprints to identify which one provides the most accurate and functional representation of the WUI for risk management purposes. Additionally, to assist in interpreting our results, we also created a WUI map based on the World Settlement Footprint. Our hypothesis is that Microsoft’s data, while highly precise, may still produce anomalies in identifying interface areas. Some detected structures might not be real priorities for protection during wildfires, leading to an overestimation of critical WUI zones. This could result in misallocated resources—both financial and operational—for fire suppression and mitigation efforts. We hope to provide insights into the reliability of the databases used for WUI mapping, highlighting the impacts of methodological choices on wildfire risk management. The results may assist policymakers and researchers in selecting more suitable databases for WUI fire analysis, as well as contribute to improving fire prevention and mitigation strategies in Portugal and elsewhere.

## Results

### Building data in Portugal

The two databases used for Wildland-Urban Interface (WUI) mapping that provide the location of buildings (Portuguese Geographic Building Database^51^(BGE21) and Microsoft’s Global Building Footprints^37^(MSB24)) show distinct features. According to BGE21, mainland Portugal comprises a total of 3,386,084 buildings, which accommodate 5,736,734 dwellings. In contrast, the MSB24 database records 5,654,476 buildings; however, it does not specify their intended utilisation (e.g., residential, industrial, or support structures). The MSB24 dataset contains approximately 67% more buildings than BGE21, though this disparity is not uniformly distributed across the country. Additionally, we also used the World Settlement Footprint^52^(WSF19) as a reference for building locations, which provides information on global human settlement. However, since it is a binary raster (built-up vs. non-built-up areas), this dataset does not allow us to count the number of buildings in the study area.

**Fig. 1:**
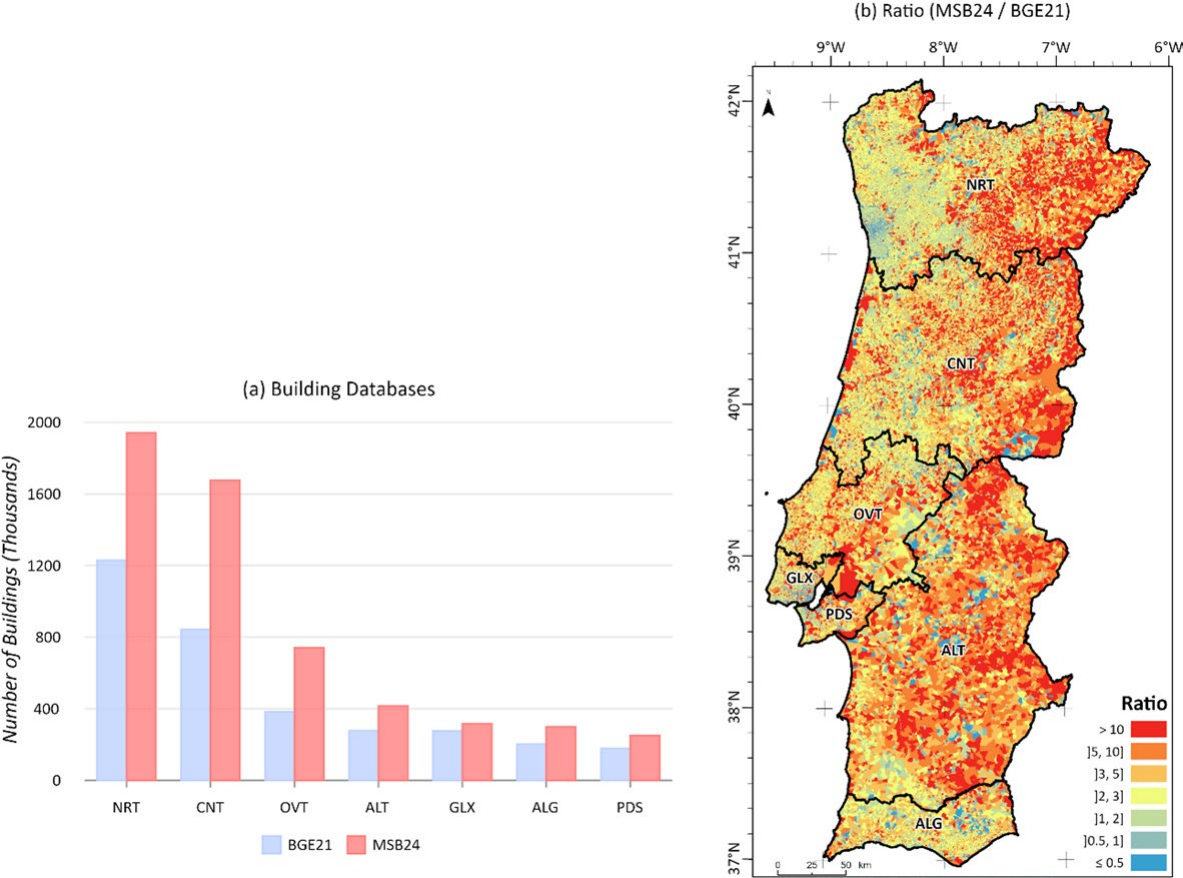
Total number of buildings by NUTS-2 region (**a**); Ratio of MSB24 buildings to BGE21 buildings by census tract (**b**). In (**b**) values above 1 indicate census tracts where there are more buildings in the Microsoft database. The NUTS II abbreviations represent: NRT: Norte; CNT: Centro; OVT: Oeste e Vale do Tejo; GLX: Grande Lisboa; PDS: Península de Setúbal; ALT: Alentejo; and ALG: Algarve. Maps were generated by the authors using ArcGIS Pro version 3.6 (https://www.esri.com).

As illustrated in Fig. 1a, the total number of buildings by NUTSII region reveals that the MSB24 dataset’s superiority in building counts is particularly pronounced in the Norte, Centro, and Oeste e Vale do Tejo regions. Furthermore, within these regions, there are marked discrepancies in building counts between census tracts. Notably, census tracts in the eastern interior exhibit higher ratios of MSB24 buildings to BGE21 buildings compared to those in coastal zones and metropolitan areas, where most of the population resides (see Figs. 1b and 4a).

Out of a total of 203,264 census tracts, MSB24 records more buildings in 149,411 (73%). The difference in the number of buildings ranged from a single unit to 548.5 times more buildings in MSB24 within a single census tract. Notably, in 87,036 (43%) of the tracts, the number of buildings was at least twice as high (ratio ≥ 2.0). Conversely, in 47,322 (23%) of the census tracts, there are more buildings in BGE21, with ratios ranging between 0.001 and 0.99. Values close to zero indicate tracts where no buildings are recorded in MSB24, as observed in some areas in the far south of Algarve. Among these, in 22,150 (47%) of cases, the number of buildings in BGE21 was twice as high, resulting in ratios lower than 0.5. In the remaining tracts, either both datasets show an equal number of buildings (3%) or no buildings at all (1%).

### WUI mapping

Following established methodologies (see “Mapping the WUI” Section), we distinguished between two WUI types: *Intermix*, where buildings and wildland vegetation are intermingled, characterized by a building density > 6.17 buildings/km^2^ and > 50% combustible cover within a 100 m radius; and *Interface*, where urban areas are located near—but not within—extensive wildland vegetation patches (> 5 km^2^), with building density > 6.17 buildings/km^2^ and < 50% combustible cover within a 100 m radius (and within 600 m of large wildland patches). The WUI maps generated with different building data for mainland Portugal showed that the total WUI area varied substantially depending on the dataset used. The total WUI area ranged from approximately 7,620 km^2^ to 13,175 km^2^ - accounting for 8.5% and 14.8% of the study area, respectively (Table 1). *Intermix* WUI areas were less dispersed across the territory compared to *Interface* WUI. Among the datasets, WUI-MSB24 identified the largest extent of WUI areas, followed by the WUI-WSF19. In contrast, WUI-BGE21 generated the smallest WUI area.

**Table 1:** WUI Area Estimates in Mainland Portugal. Maps were generated with different datasets: WUI-BGE21^51^, WUI-MSB24^37^, WUI-WSF19^52^.

Data source	WUI area (km^2^)	Intermix (%)	Interface (%)	Total WUI (%)
WUI-BGE21	7,620.70	2.7	5.8	8.5
WUI-MSB24	13,177.47	6.2	8.6	14.8
WUI-WSF19	8,899.70	3.4	6.6	10.0

**Fig. 2:**
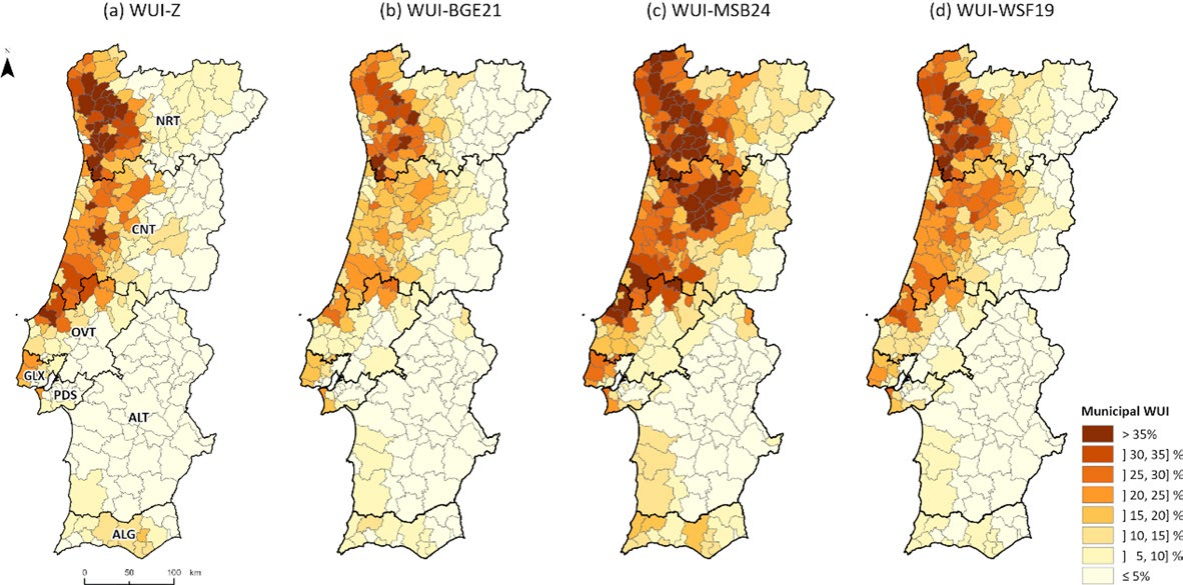
WUI distribution at municipal level in mainland Portugal. NUTS-2 abbreviations represent: NRT: Norte; CNT: Centro; OVT: Oeste e Vale do Tejo; GLX: Grande Lisboa; PDS: Península de Setúbal; ALT: Alentejo; and ALG: Algarve. (**a**) WUI-Z mapped by Bar-Massada et al.^53^, (**b**) WUI-BGE21^51^, (**c**) WUI-MSB24^37^ and (**d**) WUI-WSF19^52^. Maps were generated by the authors using ArcGIS Pro version 3.6 (https://www.esri.com).

We observed variations in the spatial distribution of WUI proportions at the municipal level, particularly along the coastal regions of the North and Centre. Maps (b), (c) and (d) in Fig. 2 show consistent patterns, with higher WUI proportions concentrated near the Northern-Central coast.

However, the WUI-MSB24 (Fig. 2c) displays notable discrepancies compared to others, especially in the Centre region, where 23 municipalities exceeded 30% WUI coverage. This highlights the significant impact of dataset selection on WUI identification.

The correspondence between different WUI mappings is shown in Table 2. Given the class imbalance in WUI mapping, particularly the dominance of the ‘Not WUI’ category, global accuracy can be misleading^54^. Therefore, we relied on the Kappa Index (KI) and the Jaccard Index (JI), which are considered more robust alternatives for thematic map accuracy assessment under class imbalance conditions^55^. While the JI quantifies the spatial overlap between classified WUI areas (i.e., the intersection over union), the KI accounts for the expected agreement due to chance, considering the distribution of all classes. Across all comparisons, JI and KI values were consistently lower than overall accuracy, reinforcing the disproportionate influence of the majority class.

Among the evaluated sources, the WUI-WSF19 showed the highest agreement with the WUI-BGE21, as evidenced by the highest JI and KI values. In contrast, although WUI-MSB24 exhibited high recall rates—especially in detecting WUI areas—its precision was notably lower, resulting in the lowest agreement indices between all evaluated datasets. This indicates substantial overestimation of WUI areas by WUI-MSB24 and reflects greater divergence in boundary delineation.

Substantial variation was observed in the consistency of the *Intermix* and *Interface* classification across mappings. *Interface* areas demonstrated consistently higher agreement among all data sources and performance metrics, while *Intermix* areas exhibited substantially lower agreement.

**Table 2:** Pairwise agreement of WUI Mapping. WUI-Z mapped by Bar-Massada et al.^53^, WUI-BGE21^51^, WUI-MSB24^37^ and WUI-WSF19^52^. These metrics quantify agreement between WUI maps and do not represent absolute accuracy, as no ground truth dataset is available for Portugal.

Data source	Compare data	Class	Precision	Recall	F1 score	Accuracy	Kappa index	Jaccard index
WUI-Z	WUI-BGE21	*Not WUI*	0.96	0.97	0.97	0.92	0.52	0.85
		*Intermix*	0.44	0.28	0.34			
		*Interface*	0.45	0.55	0.49			
	WUI-MSB24	*Not WUI*	0.98	0.92	0.95	0.88	0.45	0.79
		*Intermix*	0.27	0.39	0.32			
		*Interface*	0.36	0.64	0.46			
	WUI-WSF19	*Not WUI*	0.98	0.97	0.98	0.93	0.63	0.88
		*Intermix*	0.55	0.43	0.48			
		*Interface*	0.47	0.66	0.55			
WUI-BGE21	WUI-MSB24	*Not WUI*	1.00	0.93	0.96	0.94	0.70	0.88
		*Intermix*	0.43	0.97	0.60			
		*Interface*	0.67	0.99	0.80			
	WUI-WSF19	*Not WUI*	0.99	0.97	0.98	0.96	0.77	0.92
		*Intermix*	0.60	0.73	0.66			
		*Interface*	0.80	0.91	0.85			
WUI-WSF19	WUI-MSB24	*Not WUI*	0.99	0.94	0.96	0.93	0.71	0.88
		*Intermix*	0.46	0.84	0.59			
		*Interface*	0.73	0.95	0.82			

**Fig. 3:**
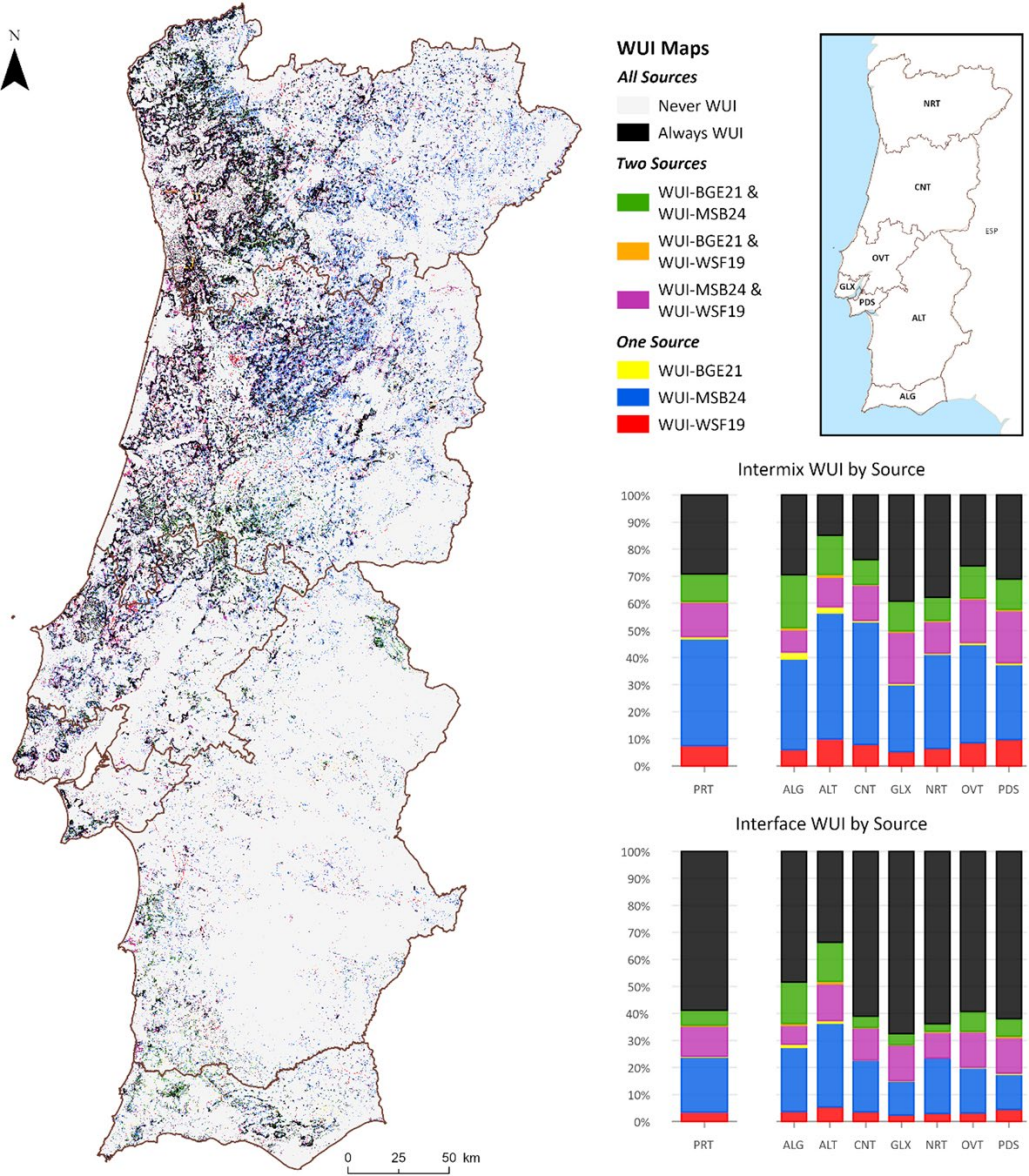
Spatial Agreement Analysis of WUI Maps. PRT: Portugal. NUTS II abbreviations represent: NRT: Norte; CNT: Centro; OVT: Oeste e Vale do Tejo; GLX: Grande Lisboa; PDS: Península de Setúbal; ALT: Alentejo; and ALG: Algarve. A more detailed visualization of these spatial discrepancies is available in the Supplementary Materials (Figure S1). Maps were generated with different datasets: WUI-BGE21^51^, WUI-MSB24^37^ and WUI-WSF19^52^. Maps were generated by the authors using ArcGIS Pro version 3.6 (https://www.esri.com).

To complement the quantitative comparison between WUI maps, Fig. 3 presents a spatial and regional overview of the agreement among the WUI mapped. The map classifies areas based on the number of sources that designate them as WUI: agreement across all three datasets, agreement between two sources, or identification by only one source. Nationally, approximately 46% of the areas classified as WUI were identified by all three datasets. However, these fully overlapping areas are sparse and restricted to specific zones, particularly along the western coastal zones and parts of the Algarve region in the south. Areas with agreement between two sources (e.g., WUI-BGE21 & WUI-MSB24 in green, WUI-BGE21 & WUI-WSF19 in yellow; see Fig. 3) are more widespread and reflect moderate consistency across datasets. Conversely, the extensive presence of WUI areas detected by only one source — especially by WUI-MSB24 (blue; see Fig. 3) — highlights methodological differences and varying sensitivities in delineating the WUI.

The accompanying bar plots (Fig. 3) further disaggregate this pattern at both national and NUTSII levels, separately for the I*ntermix* and *Interface* classes. The *Intermix* class exhibited greater variability in agreement across datasets. At the national level, only 29% of *Intermix* areas were consistently identified by all three datasets, while 47% were detected by just one source - predominantly by WUI-MSB24 (39.5%) and marginally by WUI-WSF19 (7.5%). In the Alentejo (ALT) and Centro (CNT) regions, single-source identification surpassed 50%, reinforcing the low degree of spatial overlap. Except for Algarve and Alentejo, agreement between WUI-MSB24 and WUI-WSF19 was higher than any other two-source combination in all remaining regions. In all NUTSII areas, full agreement across the three datasets in identifying *Intermix*-type WUI remained below 40%.

In contrast, the *Interface* class showed higher consistency across sources. Nationally, about 60% of *Interface* areas were jointly identified by all three datasets, with Alentejo being the only region where this agreement fell below 50%. Despite this overall consistency, single-source identification still exceeded the overlap between two sources in most regions. This indicates that, although *Interface* areas tend to have more clearly defined spatial patterns and are more readily detectable, significant methodological discrepancies remain — particularly concerning spatial thresholds and structural density criteria used to define this WUI category.

### Wildfire occurrence in WUI

In addition to comparing WUI mappings, we analysed the relationship between wildfire occurrence and WUI areas in mainland Portugal, using the fire perimeters recorded from 2000 to 2023. The annual average burned area was 128,043 hectares, with extreme variability across years (Table 3). The most severe wildfire year was 2017 (557,737 ha burned), followed by 2003 (439,908 ha) and 2005 (346,352 ha). Conversely, 2008 had the lowest burned area (11,952 ha).

When focusing on WUI areas, the proportion of burned area varied significantly by interface type and mapping methodology. At *Intermix* zones the WUI-MSB24 captured the highest average proportion of burned area (7.1%), far exceeding WUI-WSF19 (2.9%) and WUI-BGE21 (2.2%). Peak values occurred in 2017 (10.6% for WUI-MSB24), 2016 and 2023 (8.3%). In the *Interface* zones WUI-MSF19 again showed the highest average proportion (3.5%), with notable peaks in 2017 (6.8%) and 2022 (5.0%). WUI-BGE21 consistently reported the lowest values (1.6%). Overall, the proportion of burned area within WUI remained relatively low, ranging from 0.4% to 10.6% (Table 3). However, this variation showed no consistent relationship with total burned area. For example: to the year of 2006 (72,681 ha burned, below the annual average) the proportion of burned area within WUI areas ranged from 3.0% (WUI-BGE21) to 10.0% (WUI-MSB24), one of the highest values in the historical series. Similarly, in 2018 (40,278 ha burned) the highest proportion of burned area for the WUI-BGE21 *Intermix* was registered, with 4.4%, twice the annual average. On the other hand, the year of 2003, one with the largest total burned area recorded (439,908.11 ha), the proportion of burned area within WUI categories did not increase proportionally. Despite the significantly high total burned area, the variation in burned area percentages within WUI was comparatively smaller. For example, the proportional burned area in *Intermix* zones was 1.6% for WUI-BGE21 and 5.1% for WUI-MSB24, while in *Interface* zones, values ranged from 1.4% (WUI-BGE21) to 3.1% (WUI-MSB24).

**Table 3:** Ratio between burned area in WUI (BAwui) and burned area (BA) total in mainland Portugal by year. Bold values highlight the highest percentages in each row (comparing source within each category). Source of data: ICNF, 2024. wui were generated with different datasets: WUI-BGE21^51^, WUI-MSB24^37^ and WUI-WSF19^52^.

Year	Burned area (ha)	Ratio (BAwui/BAtotal)					
		*Intermix*			*Interface*		
		WUI-BGE21	WUI-WSF19	WUI-MSB24	WUI-BGE21	WUI-WSF19	WUI-MSB24
2000	143,236.63	0.7%	1.6%	4.5%	0.4%	1.1%	2.2%
2001	97,595.90	**1.5%**	**2.7%**	**6.8%**	0.5%	1.2%	1.9%
2002	133,194.80	**1.7%**	**2.7%**	**6.8%**	0.7%	1.4%	2.4%
2003	439,908.11	**1.6%**	1.4%	**5.1%**	1.4%	**1.4%**	3.1%
2004	114,967.97	**1.2%**	**2.0%**	**4.1%**	0.6%	1.0%	1.8%
2005	346,352.19	**3.3%**	**4.3%**	**8.9%**	1.8%	2.4%	3.2%
2006	72,681.55	**2.2%**	**3.5%**	**7.8%**	0.8%	1.5%	2.2%
2007	38,319.64	**1.5%**	**2.4%**	**5.9%**	1.5%	2.0%	3.1%
2008	11,951.74	0.4%	**1.1%**	**3.6%**	**0.5%**	0.9%	1.8%
2009	93,196.89	**1.3%**	**1.9%**	**5.9%**	0.8%	1.3%	3.2%
2010	131,267.98	**2.1%**	**3.1%**	**7.5%**	0.6%	1.4%	2.6%
2011	80,227.25	**1.7%**	**2.7%**	**5.7%**	0.5%	0.9%	2.0%
2012	113,574.65	**2.2%**	**2.7%**	**6.8%**	1.3%	1.5%	2.5%
2013	149,245.11	**1.6%**	**3.1%**	**6.4%**	0.7%	1.9%	2.7%
2014	18,325.91	**0.9%**	**1.6%**	**4.1%**	0.4%	0.8%	2.8%
2015	56,102.68	**2.1%**	**2.7%**	**7.1%**	0.7%	1.2%	2.6%
2016	158,287.20	**3.2%**	**4.8%**	**8.3%**	0.9%	1.7%	2.4%
2017	557,737.18	3.3%	4.3%	**10.6%**	**3.8%**	**4.5%**	6.8%
2018	40,278.86	**4.4%**	**2.4%**	**7.9%**	1.7%	1.5%	3.0%
2019	40,189.54	**1.9%**	**2.1%**	**6.5%**	1.3%	1.4%	3.4%
2020	66,257.72	**1.3%**	**1.4%**	**4.8%**	0.8%	0.9%	2.9%
2021	27,372.95	**0.9%**	**1.3%**	**3.4%**	0.8%	0.9%	1.9%
2022	108,618.27	0.6%	0.7%	1.7%	**1.8%**	**2.2%**	**5.0%**
2023	34,150.97	**2.9%**	**2.5%**	**8.3%**	1.1%	1.0%	2.1%
***Average***	***128,043.40***	***2.2%***	***2.9%***	***7.1%***	*1.6%*	*2.1%*	*3.5%*

The Kruskal-Wallis tests revealed statistically significant differences in burned area across WUI mapping methodologies for both *Intermix* (χ^2^ = 13.89, *p* < 0.001) and *Interface* (χ^2^ = 11.60, *p* < 0.001) classifications. Table 4 shows that post-hoc Dunn tests with Bonferroni correction indicated distinct patterns between WUI types. For *Intermix* areas: WUI-MSB24 differed significantly from both WUI-BGE21 and WUI-WSF19. No significant difference was found between WUI-BGE21 and WUI-WSF19 (*p* = 0.600). In contrast, for *Interface* areas: significant divergence only emerged between WUI-BGE21 and WUI-MSB24 and neither dataset differed significantly from WUI-WSF19 (all with *p* > 0.09). These results show that different datasets used for WUI delineation led to different estimates of the overlap between burned areas and the WUI.

**Table 4:** Dunn’s test pairwise comparisons. Bold values highlight the significant values *p* < 0.05. WUI were generated with different datasets: WUI-BGE21^51^, WUI-MSB24^37^ and WUI-WSF19^52^.

WUI class	Comparison	Z	*P*.adj
*Intermix*	WUI-BGE21 & WUI-MSB24	-3.57	**0.0005**
	WUI-BGE21 & WUI-WSF19	-0.84	0.6002
	WUI-MSB24 & WUI-WSF19	2.72	**0.0097**
*Interface*	WUI-BGE21 & WUI-MSB24	-3.40	**0.0010**
	WUI-BGE21 & WUI-WSB19	-1.52	0.1912
	WUI-MSB24 & WUI-WSF19	1.88	0.0910

## Discussion

This study employed multiple cartographic bases to generate high-resolution mapping of the Wildland-Urban Interface (WUI) in mainland Portugal. We used Portugal’s National Building Geographic Database (WUI-BGE21), World Settlement Footprint (WUI-WSF19), and Microsoft’s Global Building Footprints (WUI-MSB24) as primary sources. The results demonstrate that under the employed methodology, the choice of building database significantly alters the mapped WUI area. Using an exclusively residential database (WUI-BGE21), we identified 7,620 km^2^ of Portuguese territory as interface zones. Conversely, when utilizing bases accounting for all structures, the mapped area ranged from 8,899 km^2^ (WUI-WSF19) to 13,177 km^2^ (WUI-MSB24). Our initial hypothesis suggested that despite Microsoft reported high accuracy (94.3% precision, 85.9% recall, and 1.4% false positives in Europe), this dataset might overestimate risk areas – particularly in WUI delineation. While previous studies, such as Bar-Massada^44^ and Carlson et al.^45^ validated its effectiveness for WUI mapping in the U.S., its reliability varies regionally. Gevaert et al.^36^ documented significantly lower accuracy metrics in the Philippines and Tanzania compared to global benchmarks.

Carlson et al.^45^ analysed the impact of errors in Microsoft’s building dataset on WUI classification, identifying that 25% of samples contained errors of omission or commission. Nevertheless, these errors had limited impact on final classifications, resulting in a WUI omission error of just 2%. Additionally, 98% of pixels classified as WUI were correct — even when using a 500-m radius and requiring ≥ 5 structures to define WUI areas. By contrast, our methodology (using a 100-m radius and only 1 structure) is more sensitive to data variations and may amplify noise in building datasets. Our results for Portugal showed that 34% of the total mapped WUI area was identified exclusively by Microsoft data. High recall values (> 0.9) when combining WUI-MSB24 with other mappings indicate that this dataset effectively covered WUI areas detected by alternative sources. However, low precision values (< 0.7) suggest other bases inadequately identified WUI-MSB24 areas. The higher agreement observed in *interface* zones compared to *intermix* zones may be attributed to the more dispersed and heterogeneous nature of the latter, which hinders uniform detection and classification across datasets, underscoring the challenges in consistently identifying diffuse settlement patterns typical of the *Intermix* WUI class. This discrepancy was expected when comparing with WUI-BGE21 data due to differing building counts, yet the lower similarity with WUI-WSF19 is surprising — given both datasets consider all building typologies. We note that as a raster dataset (even at 10 m resolution), WSF19 cannot capture small structures as effectively as MSB24. However, the overabundance of buildings in MSB24 implies potential anomalies. This overestimation directly compromises WUI delineation, particularly in rural or inland regions. The consequences are twofold: where actual buildings remain undetected, disaster support may fail to reach vulnerable households, while resources risk being diverted to areas with non-existent structures that were erroneously flagged. Barbosa et al.^56^ propose a solution focused on the Microsoft building dataset. They suggest that applying filters to prioritize residential structures—by systematically excluding anomalies such as industrial facilities, photovoltaic arrays, and transmission lines—can significantly reduce the overestimation of mapped WUI area. This refinement in data quality could be a critical step toward more accurately identifying priority areas for resource allocation, ensuring that support is directed to genuine communities at risk.

Although substantial differences exist among the WUI maps produced from each of our input datasets, our findings align with previous comparative studies that examined WUI mappings generated using divergent methodologies^13,16,43,44,47,57^. These studies, despite methodological differences, consistently identified convergent WUI zones. Similarly, our analysis — though based on varying data sources rather than mapping techniques—also reveals areas of overlap. All WUI mappings consistently highlight heightened concentration of these areas along Portugal’s Northern-Central coastal zone—aligning with established patterns found by Bar-Massada^53^. These consistently mapped zones represent critical wildfire hotspots and deserve prioritized attention, as they represent densely populated regions within wildland-urban transition areas and are therefore more exposed to wildfires.

Historical burned area records provide empirical evidence of wildfire impacts on WUI zones. Tang et al.^16^ reported a 22% global decrease in total burned area between 2005 and 2025 yet observed a 35% increase in burned area within WUI. Similarly, Tonini et al.^20^ documented rising proportions of burned WUI areas in Portugal (1990–2012), from 4% to 14%, while Argañaraz et al.^42^ found 14% of burned areas occurred within WUI in Argentina. These values, however, vary with analytical scale and WUI delineation methodologies. Although we identified no temporal trend in WUI fires, our analysis revealed that burned WUI area is weakly associated with total annual burned area. This indicates that, despite a large wildfire event resulting in extensive total burned area, there was no significant proportional increase in affected areas within WUI categories, as might be expected. These results indicate that years with smaller burned areas (e.g., 2006, 2018) can disproportionately impact WUI zones, while megafires (e.g., 2003, 2005, 2017) primarily affect non-WUI areas. This pattern suggests that during low-burn years, fires may impact interface zones due to localized factors like weather conditions, land-use changes, or targeted suppression efforts^4,6^. Crucially, WUI vulnerability depends not only on total fire extent but also on spatial concentration in high-risk years, underscoring the need for consistent WUI-focused prevention policies irrespective of nationwide fire severity forecasts.

In summary, this study demonstrates that the choice of building location dataset fundamentally reshapes Wildland-Urban Interface delineation and wildfire risk assessments in mainland Portugal. While Microsoft Building Footprints (MSB24) provides valuable global coverage, our analysis reveals it overestimates WUI area by 73% (13,177 km^2^ vs. 7,621 km^2^ for census-based home locations BGE21), disproportionately reclassifying inland municipalities like those found in Centro region as priority zones (> 30% WUI coverage). Methodologically, the 100-m radius amplified MSB24’s biases, generating false positives in rural areas through non-residential/abandoned structures - risking resource misallocation and potentially eroding trust in AI-derived geospatial products. Critically, *Intermix* WUI exhibited the highest uncertainty, with only 29% consensus across datasets versus 60% for *Interface* areas, despite *Intermix* zones’ heightened fire vulnerability. We further observed that burned area within WUI is not directly related to total fire extent: wildfire years with lower burned area (e.g., 2006, 2018) disproportionately impacted WUI zones, whereas years of megafires (e.g., 2003, 2017) primarily consumed non-WUI areas.

These findings point to the need of implementing strategic adaptations in practice. Local risk management should prioritize validated residential data (e.g., BGE21) to avoid overestimating exposure in rural/inland regions. Specifically, we advocate deploying local datasets like BGE21 for preventive planning (e.g., fuel management, defensible space programs), while reserving high-coverage global footprints like MSB24 for emergency response where structural detection is paramount. Resource allocation must account for dataset-specific biases, as MSB24’s high recall suits rapid response but its low precision undermines preventive investments. Methodological transparency about omission/commission errors in AI-derived data is equally essential. Future work should integrate multi-source validations (e.g., crowdsourced data) and refine WUI definitions to address Mediterranean dispersed settlements.

## Methodology

### Study area

**Fig. 4:**
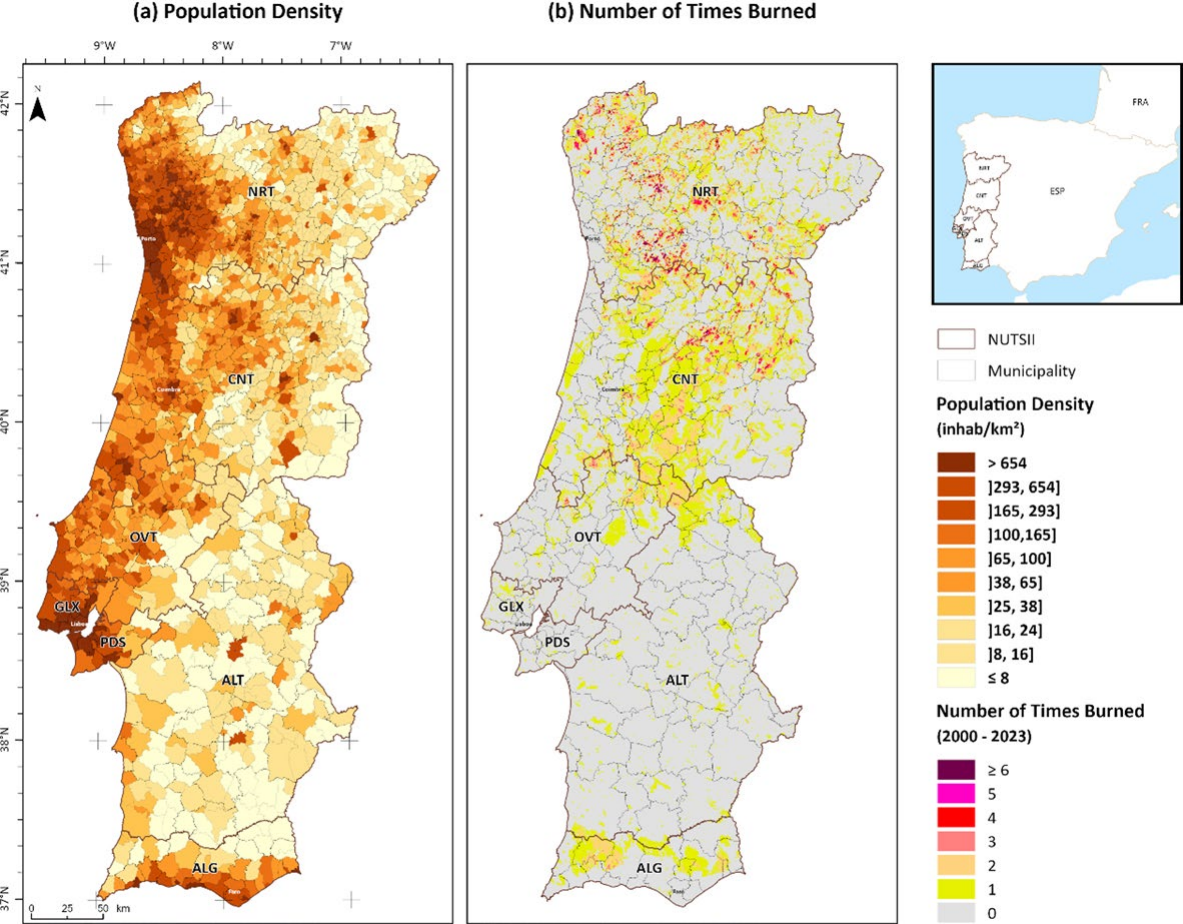
Study Area. Distribution of population density in mainland Portugal at parish level according to the 2021 Census (**a**) number of times burned between 2000–2023 based on fire perimeters (**b**). The NUTS-2 abbreviations represent: NRT: Norte; CNT: Centro; OVT: Oeste e Vale do Tejo; GLX: Grande Lisboa; PDS: Península de Setúbal; ALT: Alentejo; and ALG: Algarve. Maps were generated by the authors using ArcGIS Pro version 3.6 (https://www.esri.com).

The study area of this work is mainland Portugal, located in the southwest of the Iberian Peninsula. It is bordered by the Atlantic Ocean to the south and west, and by Spain to the north and east. According to census data, the population of Portugal was approximately 9.86 million in 2021^51^. The population distribution shows a substantially higher density in coastal areas and around the metropolitan regions of Lisbon and Porto (Fig. 4a).

According to the Portuguese Forest Services^58^, the main wildfire season typically occurs from June to September. Between 2000 and 2023, about 1,750 wildfires occurred on average every year, burning 128,043 hectares of land annually during this period. Figure 4b shows that the regions most affected by wildfires were the NUTS-2 regions of Norte, Centro, and Algarve. In 2017, Portugal experienced an extreme wildfire season driven by prolonged drought, extreme weather conditions, and human activity, resulting in over 540,000 hectares burned, more than 100 fatalities and damage to over 2,400 structures^9,59–61^. Extreme scenarios such as this are more likely to occur in the future due to the ongoing impacts of climate change^2^.

### Built-up data

With the purpose of creating the most reliable Wildland-Urban Interface (WUI) map, we assess potential discrepancies in WUI mapping generated with distinct building databases available for mainland Portugal. We selected three datasets: two vector-based identifying the exact location of individual buildings and a raster model representing the extent of built-up areas.

The Portuguese Geographic Building Database (BGE21), provided by Statistics Portugal^51^, offers vector point data representing all residential buildings with at least one housing unit at the time of the 2021 census. This dataset excludes non-residential structures (e.g., industrial, commercial or support structures), aligning with traditional WUI frameworks^11,62,63^ that prioritize human vulnerability in residential areas.

The second building dataset was obtained from Microsoft’s Global Building Footprints^37^ which includes 1.46 billion building footprints (polygon outlines encompassing all types of structures) worldwide and are in constant updating, hereafter referred to as ‘MSB24’. These footprints were identified from high-resolution satellite imagery (0.3 m) provided by Bing Maps, using a convolutional neural network semantic segmentation algorithm. The image collection dates range from 2014 to 2024. For our analysis, we converted all building footprints into points based on their centroids.

The raster dataset derived from the World Settlement Footprint^52^ provides information on global human settlement, and hereafter referred to as ‘WSF19’. This global dataset maps human settlements through AI-driven processing of 10-metre resolution satellite imagery (Sentinel-1 and Sentinel-2), with pixels categorically labelled as built-up or non-built-up areas. This dataset aggregates built-up areas at the pixel level, capturing any human-made structure without distinguishing between building types.

To identify discrepancies in building locations, we used 2021 census tracts and counted the number of buildings present in each dataset. We assumed that the buildings in MSB24 have the potential to overestimate interface areas. The distribution of resources to mitigate or prevent damage is directly associated with the number of elements at risk, and overestimating the number of structures can lead to the allocation of resources to incorrect areas^36^.

While this analysis is not feasible with WSF19 due to its binary raster format (built-up vs. non-built-up areas), this dataset can help identify areas where MSB24 may have anomalies, errors, or lower-priority zones for protection. Since both datasets include non-residential elements, the interface areas identified by them may show spatial similarities.

### Land cover data

We used the Conjunctural Land Cover Map (COSc) for mainland Portugal for the year of 2022^64,65^. It comprises 15 thematic classes at its most detailed level and has a pixel resolution of 10 m. It is produced using spatial technologies and artificial intelligence, incorporating machine learning algorithms and expert-driven rules to automatically classify multispectral and intra-annual data series derived from Sentinel-2 satellite imagery. We reclassified COSc in five thematic classes, with three considered as being combustible, namely: forest (eucalyptus, other broadleaves, maritime pine and other conifers), open forest (agroforest system - ASF, evergreen oaks and stone pine, associated with shrublands), and shrubland, whereas the other classes are classified as not combustible (grassland, artificial lands, agriculture lands, bare soil, wetland, water and ASF associated with agriculture). We did not consider grassland as a combustible material in our methodology, as it aligns with the approach used by Bar-Massada et al.^53^.

### Mapping the WUI

To generate the WUI maps for mainland Portugal using BGE21 (WUI-BGE21) and MSB24 (WUI-MSB24) data, we based our approach on previous studies that employed a point-based methodology^42,43,45,46,66,67^. To be considered a WUI candidate, the building density within a circular moving window of 100 m radius must exceed 6.17 buildings/km^2^, which corresponds to at least one building within the 100 m circular moving window. In this approach, every building has the potential to be a WUI candidate. We chose the 100 m radius because neighbourhoods of this size require fewer buildings to meet the density threshold for WUI classification^45^. This allows us to assess the discrepancies generated by the choice of datasets at a finer level of detail.

The WUI based on the WSF19 (WUI-WSF19) dataset was generated by including all cells within 100 m of a built-up cell as WUI candidates. This approach was adopted for mapping the WUI across Europe and enables the identification of all buildings exposed to wildfires^53^.

We calculated the percentage cover of combustible material within a circular moving window with a 100 m radius. Based on Radeloff et al.^11^, to identify *Intermix* WUI, we applied a threshold of combustible material cover exceeding 50% within the 100 m circular moving window, whereas for identifying *Interface-*type WUI the 100-m moving window must be less than 50% combustible material cover but located within 600 m of large combustible material patches (> 5 km^2^)^42,53^.

The generated WUI maps are in raster format with a spatial resolution of 10 m.

### WUI comparison

After mapping the WUI using each dataset, we reclassified them into WUI and Non-WUI categories to calculate the percentage of WUI area per municipality in mainland Portugal. We also assessed spatial agreement between WUI maps using pairwise comparison metrics Recall, Precision, F1-score, Accuracy, Jaccard Index, and Kappa Index^68^. It is critical to clarify the application of these metrics in the context of our study. Typically, they are used to assess a classification against a validated ground truth dataset. However, in the absence of a single authoritative WUI reference map for Portugal, we employed these metrics in a comparative framework. Each WUI map was systematically paired and compared against every other map (e.g., WUI-BGE21 vs. WUI-MSB24, WUI-BGE21 vs. WUI-WSF19). This pairwise comparison does not measure ‘accuracy’ in the conventional sense but quantifies the spatial consistency and divergence between the different methodological and data-driven outcomes. These two analyses also incorporated the mapping produced by Bar-Massada et al.^53^, hereafter referred to as ‘WUI-Z’, to verify the differences that may arise according to the datasets and methodologies adopted. This kind of comparison between the WUI generated by different datasets can demonstrate how the inclusion of all structures influences the total area and spatial patterns of the WUI^47^.

We identify the exposure of our mapped WUI to wildfires calculating the WUI area overlapping fire perimeters recorded between the years 2000 and 2023, obtained from the Portuguese Forest Services^58^. For this purpose, we converted the WUI maps to polygon format and performed an intersection with the burned area perimeters. Differences between the amount of fire-affected areas within each type of WUI were assessed using the Kruskal-Wallis test, a non-parametric method for comparing multiple independent groups. When significant differences were found, Dunn’s post-hoc pairwise comparisons with Bonferroni correction were applied to identify which dataset pairs differed significantly.

Finally, to understand the spatial distribution of discrepancies in the WUI mapped from each dataset, we combined the results. This produced a single composite map indicating four possibles scenarios: areas not identified as WUI, areas identified as WUI in all maps, regardless of the dataset used, areas mapped as WUI when using two of the datasets, and areas identified as WUI by only one dataset.

## Supplementary Information

Below is the link to the electronic supplementary material.

Supplementary Material 1

## Data Availability

The Building Footprints: Microsoft’s Global Building Footprints (MSB) (https://github.com/microsoft/GlobalMLBuildingFootprints, accessed 11/19/2024). The Portuguese Geographic Building Location: *Base Geográfica de Edifícios* (obtained by request at Statistics Portugal – *Instituto Nacional de Estatística –INE* , https://www.ine.pt/xportal/xmain?xpgid=ine_main&xpid=INE, accessed 11/19/2024). The The World Settlement Footprint 2019 (https://www.esa.int/Applications/Observing_the_Earth/Mapping_our_human_footprint_from_space, accessed 11/19/2024). The WUI maps will be made available on request.

## References

[1] San-Miguel-Ayanz, J. et al. *Forest Fires in Europe, Middle East and North Africa 2023* (Publications Office of the European Union, 2024). 10.2760/8027062.

[2] El Garroussi, S., Di Giuseppe, F., Barnard, C. & Wetterhall, F. Europe faces up to tenfold increase in extreme fires in a warming climate. *npj Clim. Atmos. Sci.* **7**, 30 (2024).

[3] Chergui, B., Fahd, S., Santos, X. & Pausas, J. G. Socioeconomic factors drive fire-regime variability in the Mediterranean basin. *Ecosystems* **21**, 619–628 (2018).

[4] Oliveira, S., Gonçalves, A. & Zêzere, J. L. Reassessing wildfire susceptibility and hazard for mainland Portugal. *Sci. Total Environ.* **762**, 143121 (2021). 33129531 10.1016/j.scitotenv.2020.143121

[5] Pausas, J. G. & Fernández-Muñoz, S. Fire regime changes in the Western Mediterranean Basin: from fuel-limited to drought-driven fire regime. *Clim. Change* **110**, 215–226 (2012).

[6] Tedim, F. et al. Defining extreme wildfire events: Difficulties, challenges, and impacts. *Fire* **1**, 9 (2018).

[7] Meier, S., Elliott, R. J. R. & Strobl, E. The regional economic impact of wildfires: Evidence from Southern Europe. *J. Environ. Econ. Manag.* **118**, 102787 (2023).

[8] Metallinou, M. M. & Log, T. Health impacts of climate change-induced subzero temperature fires. *IJERPH* **14**, 814 (2017). 28726752 10.3390/ijerph14070814 PMC5551252

[9] Ribeiro, L. M., Rodrigues, A., Lucas, D. & Viegas, D. X. The impact on structures of the pedrógão grande fire complex in June 2017 (Portugal). *Fire* **3**, 1–22 (2020).

[10] Bento-Gonçalves, A. & Vieira, A. Wildfires in the wildland-urban interface: Key concepts and evaluation methodologies. *Sci. Total Environ.* **707**, 135592 (2020). 31767309 10.1016/j.scitotenv.2019.135592

[11] Radeloff, V. C. et al. The wildland–urban interface in the United States. *Ecol. Appl.* **15**, 799–805 (2005). 10.1002/eap.2597 35340097

[12] Alexandre, P. M. et al. The relative impacts of vegetation, topography and spatial arrangement on building loss to wildfires in case studies of California and Colorado. *Landsc. Ecol.* **31**, 415–430 (2016).

[13] Li, S., Dao, V., Kumar, M., Nguyen, P. & Banerjee, T. Mapping the wildland-urban interface in California using remote sensing data. *Sci. Rep.* **12**, 5789 (2022). 10.1038/s41598-022-09707-7 PMC8987053 35388077

[14] Radeloff, V. C. et al. Rapid growth of the US wildland-urban interface raises wildfire risk. *Proc. Natl. Acad. Sci. U. S. A.* **115**, 3314–3319 (2018). 29531054 10.1073/pnas.1718850115 PMC5879688

[15] Syphard, A. D. & Keeley, J. E. Factors associated with structure loss in the 2013–2018 California wildfires. *Fire* **2**, 1–15 (2019).

[16] Tang, W., He, C., Emmons, L. & Zhang, J. Global expansion of wildland-urban interface (WUI) and WUI fires: Insights from a multiyear worldwide unified database (WUWUI). *Environ. Res. Lett.* **19**, 044028 (2024).

[17] Schug, F. et al. The global wildland–urban interface. *Nature*10.1038/s41586-023-06320-0 (2023). 37468636 10.1038/s41586-023-06320-0 PMC10482693

[18] Guo, Y., Wang, J., Ge, Y. & Zhou, C. Global expansion of wildland-urban interface intensifies human exposure to wildfire risk in the 21st century. *Science Advances* **10**, eado9587 (2024). 10.1126/sciadv.ado9587 PMC11546813 39514665

[19] Godoy, M. M., Martinuzzi, S., Masera, P. & Defossé, G. E. Forty years of wildland urban interface growth and its relation with wildfires in Central-Western Chubut, Argentina. *Front. Glob Change*. **5**, 850543 (2022).

[20] Tonini, M., Parente, J. & Pereira, M. G. Global assessment of rural-urban interface in Portugal related to land cover changes. *Nat. Hazards Earth Syst. Sci.* **18**, 1647–1664 (2018).

[21] Taylor, L. No boundaries: Exurbia and the study of contemporary urban dispersion. *GeoJournal* **76**, 323–339 (2011).

[22] Badia, A., Pallares-Barbera, M., Valldeperas, N. & Gisbert, M. Wildfires in the wildland-urban interface in Catalonia: Vulnerability analysis based on land use and land cover change. *Sci. Total Environ.* **673**, 184–196 (2019). 30986678 10.1016/j.scitotenv.2019.04.012

[23] Bowman, D. M. J. S., O’Brien, J. A. & Goldammer, J. G. Pyrogeography and the global quest for sustainable fire management. *Annu. Rev. Environ. Resour.* **38**, 57–80 (2013).

[24] Nunes, A. N. Regional variability and driving forces behind forest fires in Portugal an overview of the last three decades (1980–2009). *Appl. Geogr.* **34**, 576–586 (2012).

[25] Bar-Massada, A., Radeloff, V. C. & Stewart, S. I. Biotic and abiotic effects of human settlements in the wildland–urban interface. *BioScience* **64**, 429–437 (2014).

[26] Chas-Amil, M. L., Touza, J. & García-Martínez, E. Forest fires in the wildland-urban interface: A spatial analysis of forest fragmentation and human impacts. *Appl. Geogr.* **43**, 127–137 (2013).

[27] Lampin-Maillet, C. et al. Mapping wildland-urban interfaces at large scales integrating housing density and vegetation aggregation for fire prevention in the South of France. *J. Environ. Manag.* **91**, 732–741 (2010). 19879685 10.1016/j.jenvman.2009.10.001

[28] Syphard, A. D. et al. Human influence on California fire regimes. *Ecol. Appl.* **17**, 1388–1402 (2007). 17708216 10.1890/06-1128.1

[29] Cutter, S. L., Boruff, B. J. & Shirley, W. L. Social vulnerability to environmental hazards. *Soc. Sci. Q.* **84**, 242–261 (2003).

[30] Wisner, B., Blaikie, P. M., Cannon, T. & Davis, I. *At Risk: Natural Hazards, People’s Vulnerability and Disasters* (Routledge - Taylor & Francis Group, 2004).

[31] Fernández-García, V., Beltrán-Marcos, D. & Calvo, L. Building patterns and fuel features drive wildfire severity in wildland-urban interfaces in Southern Europe. *Landsc. Urban Plann.* **231**, 104646 (2023).

[32] Syphard, A. D., Keeley, J. E., Massada, A. B., Brennan, T. J. & Radeloff, V. C. Housing arrangement and location determine the likelihood of housing loss due to wildfire. *PLoS ONE* **7**, e33954 (2012). 10.1371/journal.pone.0033954 PMC3314688 22470499

[33] Price, O. F. & Bradstock, R. A. The spatial domain of wildfire risk and response in the wildland urban interface in Sydney, Australia. *Nat. Hazards Earth Syst. Sci.* **13**, 3385–3393 (2013).

[34] Scarpa, C. et al. Modelling wildfire activity in wildland–urban interface (WUI) areas of Sardinia, Italy. *Int. J. Wildland Fire* **33**, WF24109 (2024).

[35] Oliveira, S. et al. Assessing risk and prioritizing safety interventions in human settlements affected by large wildfires. *Forests* **11**, 859 (2020).

[36] Gevaert, C. M., Buunk, T. & Van Den Homberg, M. J. C. Auditing geospatial datasets for biases: Using global building datasets for disaster risk management. *IEEE J. Sel. Top. Appl. Earth Obs. Remote Sens.* **17**, 12579–12590 (2024).

[37] Microsoft. Global ML buildings footprints (2024).

[38] Boo, G. et al. High-resolution population estimation using household survey data and building footprints. *Nat. Commun.* **13**, 1330 (2022). 35288578 10.1038/s41467-022-29094-x PMC8921279

[39] Jongman, B., Ward, P. J. & Aerts, J. C. J. H. Global exposure to river and coastal flooding: Long term trends and changes. *Glob. Environ. Change*. **22**, 823–835 (2012).

[40] Kooshki, M. et al. Towards a global impact-based forecasting model for tropical cyclones. *Nat. Hazards Earth Syst. Sci.* **24**, 309–329 (2024).

[41] Marty, R. & Duhaut, A. Global poverty estimation using private and public sector big data sources. *Sci. Rep.* **14**, 3160 (2024). 38326444 10.1038/s41598-023-49564-6 PMC10850149

[42] Argañaraz, J. P. et al. Assessing wildfire exposure in the wildland-urban interface area of the mountains of central Argentina. *J. Environ. Manag.* **196**, 499–510 (2017). 28347968 10.1016/j.jenvman.2017.03.058

[43] Barbosa, B., Oliveira, S., Caetano, M. & Rocha, J. Mapping the wildland-urban interface at municipal level for wildfire exposure analysis in mainland Portugal. *J. Environ. Manag.* **368**, 122098 (2024). 39126844 10.1016/j.jenvman.2024.122098

[44] Bar-Massada, A. A comparative analysis of two major approaches for mapping the wildland-urban interface: A case study in California. *Land* **10**, 679 (2021).

[45] Carlson, A. R., Helmers, D. P., Hawbaker, T. J., Mockrin, M. H. & Radeloff, V. C. The wildland–urban interface in the United States based on 125 million building locations. *Ecol. Appl.* **32**, e2597 (2022). 10.1002/eap.2597 35340097

[46] Kaim, D., Helmers, D. P., Jakiel, M., Pavlačka, D. & Radeloff, V. C. The wildland-urban interface in Poland reflects legacies of historical national borders. *Landscape Ecol.* **38**, 2399–2415 (2023).

[47] Ketchpaw, A. R. et al. Using structure location data to map the wildland–urban interface in Montana, USA. *Fire* **5**, 129 (2022).

[48] Gevaert, C. M., Carman, M., Rosman, B., Georgiadou, Y. & Soden, R. Fairness and accountability of AI in disaster risk management: Opportunities and challenges. *Patterns* **2**, 100363 (2021). 34820647 10.1016/j.patter.2021.100363 PMC8600248

[49] Masinde, B. et al. Auditing flood vulnerability geo-intelligence workflow for biases. *IJGI* **13**, 419 (2024).

[50] Decree-Law No. 82/2021, of 13 October. https://www.pgdlisboa.pt/leis/lei_mostra_articulado.php?nid=3453&tabela=leis&so_miolo=.

[51] INE. Census 2021 - XVI Population Census. VI Housing Census (2022).

[52] Marconcini, M. et al. Outlining where humans live, the world settlement footprint 2015. *Sci. Data* **7**, 242 (2020). 32686674 10.1038/s41597-020-00580-5 PMC7371630

[53] Bar-Massada, A., Alcasena, F., Schug, F. & Radeloff, V. C. The wildland – urban interface in Europe: Spatial patterns and associations with socioeconomic and demographic variables. *Landsc. Urban Plann.* **235**, 104759 (2023).

[54] Congalton, R. G. A review of assessing the accuracy of classifications of remotely sensed data. *Remote Sens. Environ.* **37**, 35–46 (1991).

[55] Foody, G. M. Status of land cover classification accuracy assessment. *Remote Sens. Environ.* **80**, 185–201 (2002).

[56] Barbosa, B., Oliveira, S. & Rocha, J. Semi-automated multi-criteria filtering of building footprints for enhanced wildland-urban interface mapping in mainland Portugal. *Data Brief*. 10.1016/j.dib.2025.112055 (2025). 10.1016/j.dib.2025.112055 PMC12477925 41030659

[57] Stewart, S. I. et al. Wildland-urban interface maps vary with purpose and context. *J. Forest.* **107**, 78–83 (2009).

[58] ICNF. Burned Area (2000–2023) (2024).

[59] Guerreiro, J. et al. *Avaliação Dos Incêndios Ocorridos Entre 14 e 16 de Outubro de 2017 Em Portugal Continental*. https://www.parlamento.pt/Documents/2018/Marco/RelatorioCTI190318N.pdf (2018).

[60] Ramos, A. M. et al. The compound event that triggered the destructive fires of October 2017 in Portugal. *iScience* **26**, 106141 (2023). 10.1016/j.isci.2023.106141 PMC10006635 36915678

[61] Turco, M. et al. Climate drivers of the 2017 devastating fires in Portugal. *Sci. Rep.* **9**, 13886 (2019). 31601820 10.1038/s41598-019-50281-2 PMC6787010

[62] Caggiano, M. D., Hawbaker, T. J., Gannon, B. M. & Hoffman, C. M. Building loss in WUI disasters: Evaluating the core components of the wildland–urban interface definition. *Fire* **3**, 73 (2020).

[63] Calkin, D. E., Cohen, J. D., Finney, M. A. & Thompson, M. P. How risk management can prevent future wildfire disasters in the wildland-urban interface. *Proc. Natl. Acad. Sci. U. S. A.* **111**, 746–751 (2014). 10.1073/pnas.1315088111 PMC3896199 24344292

[64] Costa, H., Benevides, P., Moreira, F. D., Moraes, D. & Caetano, M. Spatially stratified and multi-stage approach for national land cover mapping based on sentinel-2 data and expert knowledge. *Remote Sens.* **14**, 1865 (2022).

[65] DGT. Carta de Uso e Ocupação do Solo (COS) (2025).

[66] Bar-Massada, A., Stewart, S. I., Hammer, R. B., Mockrin, M. H. & Radeloff, V. C. Using structure locations as a basis for mapping the wildland urban interface. *J. Environ. Manag.* **128**, 540–547 (2013). 23831676 10.1016/j.jenvman.2013.06.021

[67] Tikotzki, I., Bar-Massada, A. & Levin, N. A geographically flexible approach for mapping the wildland-urban interface integrating fire activity data. *Front. Environ. Sci.* **11**, 1231490 (2023).

[68] Pedregosa, F. et al. Scikit-learn: Machine learning in Python. *J. Mach. Learn. Res.* **12**, 2825–2830 (2011).

