# Supplementary material for: Assessing the influence of building data choice on wildland–urban interface delineation in mainland Portugal
Source: Sci Rep. 2026 May 21;16:25069. doi: 10.1038/s41598-026-53590-5 (PMC13462319; doi:10.1038/s41598-026-53590-5)
Supplement: Supplementary file 1 [file 41598_2026_53590_MOESM1_ESM.docx]

# Supplementary Material

Figure S1 – Zoom to the spatial Agreement Analysis of WUI Maps. Detailed view of Mangualde municipality (a); Orthophoto from 2023 (b); the three reference data used to map the WUI (c); and the differences between the WUI maps (d), where areas consistently mapped as WUI are shown in black, areas classified as WUI only with MSB24 data are in blue, and areas mapped with both MSB24 and WSF19 are in pink. Maps were generated by the authors using ArcGIS Pro version 3*.6* (https://www.esri.com).
